# Randomized Controlled Trial of Transcranial Direct Current Stimulation over the Supplementary Motor Area in Tourette Syndrome

**DOI:** 10.1002/mdc3.14285

**Published:** 2024-11-29

**Authors:** Yasamin Mahjoub, Natalia Szejko, Liu Shi Gan, Janet Adesewa Adeoti, Michael A. Nitsche, Carmelo M. Vicario, Tamara M. Pringsheim, Davide Martino

**Affiliations:** ^1^ Department of Clinical Neurosciences, Cumming School of Medicine University of Calgary Calgary Canada; ^2^ Department of Bioethics Medical University of Warsaw Warsaw Poland; ^3^ Hotchkiss Brain Institute, University of Calgary Calgary Canada; ^4^ Department of Psychology and Neurosciences Leibniz Research Centre for Working Environment and Human Factors Dortmund Germany; ^5^ University Clinic of Psychiatry and Psychotherapy and University Clinic of Child and Adolescent Psychiatry and Psychotherapy, Protestant Hospital of Bethel Foundation, University Hospital OWL, Bielefeld University Bielefeld Germany; ^6^ Department of Cognitive Sciences, Psychology, Education and Cultural Studies University of Messina Messina Italy; ^7^ Department of Clinical Neurosciences, Psychiatry, Pediatrics, and Community Health Sciences University of Calgary Calgary Canada

**Keywords:** transcranial direct current stimulation, Tourette syndrome, noninvasive brain stimulation, supplementary motor area, tic severity

## Abstract

**Background:**

Transcranial direct current stimulation (tDCS) over the supplementary motor area (SMA) has shown promise in Tourette syndrome (TS), but previous studies were limited in size and stimulation duration.

**Objective:**

The aim was to explore the efficacy and safety of multiple sessions of cathodal tDCS over the bilateral SMA on tic severity in TS.

**Methods:**

A double‐blind, randomized, sham‐controlled trial 1 mA cathodal tDCS over bilateral SMA was performed in participants with TS older than 16 years. The intervention involved two 20‐min periods of stimulation with either sham or active tDCS per day, over 5 consecutive days, during which participants actively suppressed tics. Tic severity was measured using the Yale Global Tic Severity Scale Total Tic Severity (YGTSS‐TTS, primary outcome) score at baseline, day 5 (visit 5), and 1 week later (visit 6). Questionnaires focusing on comorbidities were performed at baseline and visit 6.

**Results:**

Twenty‐four participants were randomly assigned (12 active, 12 sham; 8 women; median age: 26). We observed a significant effect of visit on YGTSS‐TSS, but no significant effect of treatment or treatment × visit interaction emerged. In contrast, a statistically significant effect of the treatment × visit interaction was observed for the motor tic subscore, with significantly larger improvement in the active arm. Furthermore, we detected a significantly larger decrease in premonitory urge intensity at visit 6 after active stimulation. No effect was detected on severity of comorbidities.

**Conclusions:**

This preliminary study suggests that bilateral tDCS over the SMA provides small, but significant benefits in reducing motor tic severity in TS.

Therapeutic strategies for Tourette syndrome (TS) are behavioral and pharmacological.^1^, ^2^ However, a proportion of patients benefit partially from these approaches due to limited efficacy and tolerability.^3^ Deep brain stimulation is an option in severe cases but is not readily available in many geographical areas.^4^, ^5^ Therefore, new treatment options, ideally combining good tolerability, efficacy, and ease of access, are needed.

Noninvasive brain stimulation (NIBS) has been tested in TS using different modalities and targets.^6^, ^7^ Cortical targets were selected based on a conceptualization of tics as prepotent actions modulated by inhibitory control that increases in efficiency during development.^8^, ^9^ NIBS relies on the generation of plastic rearrangements of cerebral regions and networks. Neural hubs involved in generating actions and urges to act, such as medial frontal cortical areas like the supplementary motor area (SMA), are promising NIBS targets in TS. The SMA appears to be hyperexcitable in patients with TS immediately before tic onset.^10^ Increased connectivity between SMA and basal ganglia^11^ and enhanced functional coupling between SMA and M1 during premovement/movement phases of both tics and self‐paced finger movements^12^, ^13^, ^14^ have been reported in TS. This supports the rationale for a therapeutic application of inhibitory modulation of the SMA in TS.

The most extensively explored NIBS modality in TS has been inhibitory transcranial magnetic stimulation (TMS), either as 1‐Hz repetitive TMS^15^ or as continuous θ burst stimulation,^16^ which has been applied to the SMA, M1, premotor cortex, and parietal regions in case series^6^, ^7^ and sham‐controlled trials^15^, ^16^ with heterogeneous results. Electrical current stimulation strategies provide advantages compared to TMS, including applicability in a home environment and, as a result, larger practicability. In transcranial direct current stimulation (tDCS), a low‐voltage current runs between 2 or more electrodes, with 1 electrode positioned on the scalp over the target area in standard arrangements. According to a general principle, cathodal tDCS is thought to suppress resting membrane potential (inhibitory), whereas the reverse is true for anodal tDCS, although in reality effects may not always follow this dichotomy, particularly on nonmotor domains.^17^, ^18^ Studies exploring inhibitory tDCS in TS have targeted the pre‐SMA/SMA (with the cathode positioned over the scalp corresponding to this region), but the number of participants has been very small so far, and study designs have varied. The only sham‐controlled randomized trial of SMA tDCS was a single‐session, cross‐over trial that did not detect a significant interaction between pre‐/post‐intervention time points and real/sham conditions.^19^ Several methodological limitations of tDCS studies in TS, for example, low number of sessions or sample size, unclear brain activity state of participants during stimulation (ie, actively suppressing tics or “at rest”), and limited outcome measures, prevent clear conclusions on its therapeutic potential in TS.^6^

Here, we present a proof‐of‐principle, double‐blind, parallel‐group, sham‐controlled randomized trial testing the efficacy of 1‐mA cathodal tDCS over the SMA in individuals with TS older than 16 years. The polarity of the stimulation followed the general principle interpreting cathodal tDCS effects as inhibitory. To tackle the limitations of previous studies, we aimed to (1) enroll a naturalistic and representative sample of TS patients; (2) apply an intensified protocol of repeated sessions over 5 consecutive days, in line with evidence showing that repeated tDCS may be more effective in inducing neuroplasticity (although less is known about optimal intervals^20^, ^21^, ^22^) and the notion that another modality, repetitive TMS (rTMS) of SMA, exhibits greater efficacy with a higher number of sessions^15^; (3) replace the rest condition with an active suppression mode, to improve the homogeneity of brain activity during stimulation sessions and guide tDCS effects by TS‐related brain activity (ie, promoting synergistic effects); and (4) select secondary outcome measures, including video‐based tic rating measures.

## Patients and Methods

### Study Design and Participants

This was a double‐blind, randomized, sham‐controlled, single‐center pilot trial (registered at ClinicalTrials.gov: NCT03401996). Twenty‐four participants aged 16 or older meeting *DSM‐5* (*Diagnostic and Statistical Manual of Mental Disorders*) criteria for TS were recruited through screening performed at the Calgary Tourette Syndrome Clinic and the Movement Disorders Clinic at the University of Calgary. To meet eligibility, participants had to (1) have a rating of moderately ill or worse on the Clinical Global Impression of Severity (CGI‐S) scale and a total motor *or* vocal tic severity subscore of at least 15, or a total tic severity score greater than 22 on the Yale Global Tic Severity Rating Scale (YGTSS)^23^ at screening; (2) be on a stable medication regimen during the 3 months prior to screening; and (3) be clinically stable in any psychiatric comorbidities. Individuals with metal in the head or neck area, implantable devices, a history of epilepsy, traumatic brain injury, cardiac disease, learning disability or dyslexia, and severe vision or hearing impairment were excluded from the study.

### Intervention

**FIG. 1:**
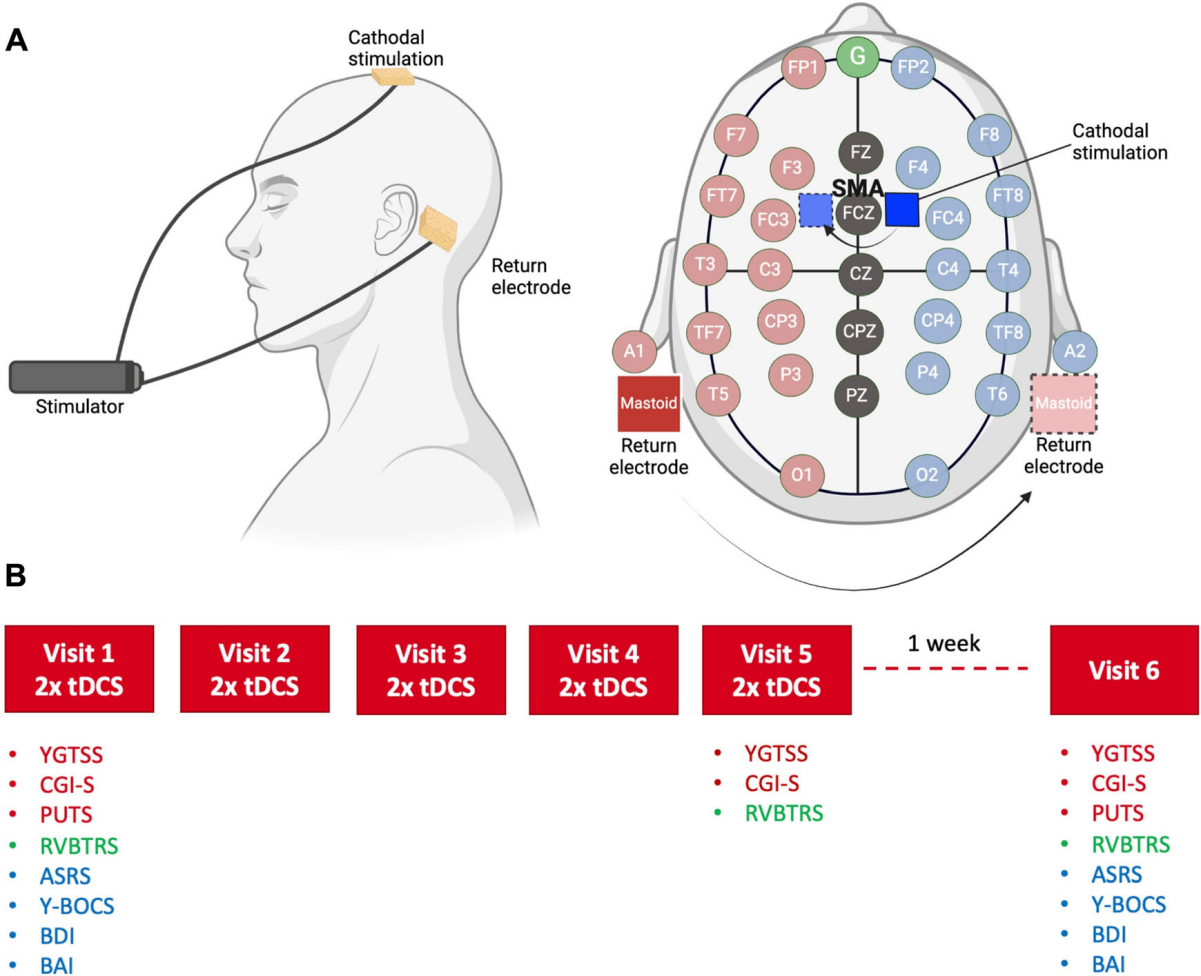
(**A**) Schematic demonstration of the position of the cathodal electrode and the anodal return electrodes. (**B**) Outline of stimulation timeline, clinical assessments, and questionnaires completed at each visit. On visit 1, the YGTSS, CGI‐S, and PUTS as well as questionnaires pertaining to comorbidities (ASRS, Y‐BOCS, BDI, and BAI) were completed. The video‐based RVBTRS was also performed, and participants subsequently underwent stimulation with real or sham tDCS. tDCS was performed daily for 5 consecutive days, and the RVBTRS was repeated at visit 3. At the end of the final stimulation session (visit 5), the YGTSS, CGI‐S, and RVBTRS were completed once again. After 1 week, participants returned for visit 6, which encompassed all outcome measures collected on visit 1. ASRS, Adult ADHD Self‐Report Scale; BAI, Beck Anxiety Inventory; BDI, Beck Depression Inventory; CGI‐S, Clinical Global Impression of Severity; PUTS, Premonitory Urge for Tics Scale; RVBTRS, Rush Video‐Based Tic Rating Scale; tDCS, transcranial direct current stimulation; Y‐BOCS, Yale‐Brown Obsessive‐Compulsive Scale; YGTSS, Yale Global Tic Severity Scale. Image created using BioRender.com.

SMA landmarking was based on the international 10 to 20 electroencephalography electrode system (Fig. 1). The FCz site was identified along the nasion‐to‐inion line, 10% of the nasion‐to‐inion distance line anterior to CZ and, based on preliminary current flow modeling, the center of the cathodal electrode was placed on the scalp 5 mm left (first 20‐min period) and 5 mm right (second 20‐min period) to FCz.^19^, ^24^, ^25^ The return electrode was placed over the right (first period) and left (second period) mastoid.^26^, ^27^ A battery‐powered constant current DC stimulator (Soterix Medical Inc., New York, USA) was used to deliver 1 mA of direct current through two 25‐cm^2^ (5 ×5 cm) saline‐soaked surface sponge electrodes.

After electrode placement, cathodal tDCS or sham stimulation was applied for 2 consecutive 20‐min‐long periods over the SMA, with no delay in between apart from the time required to switch the side of the electrode pairs. The current was ramped up to 1 mA over 30 s, maintained for 20 min, and then ramped down again over 30 s. With sham tDCS, the current was similarly ramped up to 1 mA over 30 s, which was immediately followed by a 30‐s ramp down to 0 mA. The current remained at 0 until the end of the stimulation period, where it was again ramped up to 1 mA and ramped down to 0 mA as described earlier. This sham procedure elicits identical scalp sensations as active tDCS and has demonstrated effective blinding in tDCS trials.^28^ During each stimulation session, participants were asked to actively suppress their tics while maintaining their sitting position during the whole duration of the stimulation.

Twenty of the 24 participants (10 in each group) had received a course of habit reversal training in the 2 months prior to study participation and were asked to include their learned competing motor responses in their tic‐suppressing strategies during the stimulation session. Five stimulation sessions were completed for each participant over 5 consecutive days (visits 1–5). Participants were asked to guess whether they had received cathodal or sham tDCS and completed a questionnaire to report the presence of any side effects during and in between stimulation sessions. Side effects were categorized as follows: headache or craniofacial pain, paresthesia, fatigue/daytime sleepiness, weakness, and other symptoms.

### Outcome Measures and Study Endpoints

#### Primary Outcome Measure

Figure 1B summarizes the study design. The primary outcome measure was the YGTSS‐TTS score (see Supplementary File: Data S1 for more details on rating scales for tics), and the change in YGTSS score from visits 1 to 6 was the primary endpoint of the study. Because the YGTSS‐TTS score is computed summing up motor tic and phonic tic subscores, our primary analyses also included the effect of the intervention on motor tic and phonic tic subscores separately. The YGTSS was administered by the same experienced researcher (D.M.), blinded to the randomization status, to all participants at all study visits.

#### Secondary Outcome Measures

Secondary outcome measures for tic severity included the Clinical Global Impression of Severity (CGI‐S), a generic instrument to rate symptom severity, and the Rush Video‐Based Tic Rating Scale (RVBTRS),^29^ the only validated instrument to measure current tics objectively through a video recording as well as the ability of patients to actively inhibit tics. We furthermore used the self‐report Premonitory Urge for Tics Scale (PUTS)^30^ to rate premonitory urges, as this is the most widely used and recommended validated instrument to this aim. Comorbid psychiatric disorders were measured using the following validated, self‐report rating scales: Adult ADHD Self‐Report Scale (ASRS),^31^ Yale‐Brown Obsessive‐Compulsive Scale (Y‐BOCS),^32^ Beck Depression Inventory (BDI),^33^ and Beck Anxiety Inventory (BAI).^34^

### Experimental Procedures

Participants were randomly assigned 1:1 to either sham or active tDCS groups, and allocation concealment was performed using serially numbered closed envelopes by the study coordinator, who also enrolled participants and assigned them to the study interventions. Both participants and the evaluating clinician (D.M.) were blinded to randomization status throughout the duration of the study.

Immediately before stimulation in visit 1, the examining researcher collected YGTSS and CGI‐S baseline scores; furthermore, participants completed the PUTS, ASRS, Y‐BOCS, BDI, and BAI scales. Immediately after stimulation on visit 5, YGTSS and CGI‐S were administered again. One week after the final stimulation session, participants returned (visit 6), and YGTSS, CGI‐TS, PUTS, ASRS, Y‐BOCS, BDI, and BAI were administered again. In addition to these scales, the RVBTRS was evaluated at visits 1 (before the first session), 5 (immediately after the fifth session), and 6. The protocol for the video recordings is described elsewhere.^29^ To evaluate tic suppression, an additional 5‐min video recording was added to the RVBTRS protocol (2.5 min head and shoulders, and 2.5 min whole body video) during which participants were instructed to suppress their tics as much as possible while alone during rest. The videos were evaluated independently offline by 2 clinicians (D.M. and T.M.P.) with experience in tic rating. Tic suppression was measured as inhibition potency (IP), defined as IP = RF − RI/RF, where RF is the RVBTRS score during “free” tic release recorded using the standard RVBTRS protocol and RI is the score during the tic suppression period that was recorded at the end of the standard RVBTRS protocol.^35^ The interrater agreement between the 2 expert raters was good (intraclass correlation coefficient = 0.86).

### Sample Size

Sample size calculations were based on a design with a 2‐tailed hypothesis, an α error level of 0.05, 95% confidence interval, and a β level of 0.2. The variance in the primary outcome measure was based on the standard deviation (SD = 8.8) of the YGTSS‐TTS score from the Calgary TS Clinic population, and a conservative clinically meaningful change of 9 points on the same instrument was set as critical effect. This resulted in a minimum of 12 participants per group (24 in total) to detect a significant effect on the primary outcome measure.

### Statistical Analysis

Data analysis was carried out using GraphPad Prism 9. The Shapiro–Wilk normality test confirmed normal distribution for all baseline continuous variables apart from age and disease duration. Baseline comparisons were conducted using 2‐tailed Student's *t*‐tests (normally distributed data), Mann–Whitney test (not normally distributed ordinal data), and *χ* ^2^ or Fisher's exact test (nominal data). Outcome data for YGTSS, CGI, and RVBTRS were analyzed using a repeated‐measures analysis of variance (ANOVA), or a mixed effects model in the case of missing values. The dependent variables were clinical outcome measures, and factors included the visit and treatment arm. When significant effects were found in these overall analyses, post hoc comparisons using Bonferroni‐corrected *t*‐tests were performed. Outcome data for PUTS, ASRS, Y‐BOCS, BDI, and BAI, collected only at baseline and visit 6, were analyzed for each arm using Bonferroni‐corrected 2‐tailed Student's *t*‐tests. The predetermined level of statistical significance was *P* < 0.05.

### Computational Model

To identify the electrical fields induced by the tDCS protocol in target areas, we carried out a computational model of current flow. Finite element modeling of the electric field distribution induced by tDCS was performed via the Realistic vOlumetric‐Approach‐based Simulator for Transcranial electric stimulation (ROAST) software package.^36^ Electric field magnitude was calculated in 6 regions of interest (Fig. S1A,B): (1) left and right SMA mask, with location based on the AAL3 atlas^37^; (2) left and right SMA proper, approximated by a 5‐mm‐radius sphere with the center MNI (Montreal Neurological Institute) coordinates of −3, −11, 61 and 3, –11, 61, respectively; and (3) left and right pre‐SMA, approximated by a 5‐mm‐radius sphere with the center MNI coordinates of −4, 3, 59 and 4, 3, 59, respectively.^38^ The simulation was performed based on T1‐weighted magnetic resonance imaging (MRI) scans of 7 of the 12 participants randomly assigned to the active stimulation arm.

## Results

### Baseline Characteristics

Twelve participants were randomly assigned between 2019 and 2022 to the active stimulation and 12 to the sham arm. The median age of participants was 26 years (interquartile range: 13). There was no significant difference between baseline demographic characteristics in the 2 arms (Table 1). Fifteen participants reported being uncertain about their randomization status; of the other 9, only 5 reported it correctly (active for 3 of 5); the difference in this judgment between the 2 arms was not significant (Fisher's exact test = 0.71; Table 1).

**TABLE 1:** Demographics, comorbidities, medications, and baseline scores of the participants in the sham and active stimulation groups

Demographic/clinical feature		Sham (n = 12)	Active (n = 12)	*P*‐value
Female (number)		5	3	0.67
Median age (interquartile range)		27.0 (11.5)	23.0 (16)	0.42
Median disease duration (interquartile range)		20.0 (9.5)	15.5 (15.5)	0.19
Comorbidities (number of participants)	Obsessive‐compulsive disorder	3	7	0.21
	Attention deficit hyperactivity disorder	3	2	1.00
	Anxiety and/or depressive disorder	6	5	1.00
Medications (number of participants)	SSRIs	3	4	1.00
	SNRIs	2	3	1.00
	TCAs	0	1	1.00
	Benzodiazepines	1	2	1.00
	α‐Adrenergic agonists	1	4	0.32
	Stimulants	3	4	1.00
	Antipsychotics	1	3	0.59
	Botulinum toxin	2	2	1.00
Mean baseline scores at visit 1 (±SD)	YGTSS total motor score	16.00 (2.92)	16.67 (3.65)	0.65
	YGTSS total phonic score	7.00 (4.97)	10.83 (8.10)	0.20
	YGTSS Total Tic Severity score	23.00 (6.06)	27.42 (10.77)	0.43
	CGI‐S	2.92 (0.67)	3.17 (0.58)	0.49
	PUTS	30.42 (6.19)	29.17 (6.07)	0.15
	RVBTRS	9.78 (3.15)	9.25 (3.62)	0.71
	Tic count over 10 min (5′ with examiner plus 5′ when alone in room)	30.89 (16.90)	26.83 (16.85)	0.61
	Inhibition potency	0.68 (0.29)	0.71 (0.25)	0.93
	ASRS	42.82 (13.67)	39.92 (17.60)	0.84
	Y‐BOCS	12.33 (8.56)	14.25 (7.92)	0.54
	BAI	12.50 (8.98)	16.42 (11.29)	0.40
	BDI	14.67 (9.96)	15.67 (11.19)	0.92
Participants' judgment of their randomization status	Uncertain	7	8	
	Correct	2	3	
	Incorrect	3	1	Fisher's exact test =

Age and disease duration were compared using Mann–Whitney tests, and the remainder of measures were compared using 2‐tailed Student's *t*‐tests.

Abbreviations: SSRIs, serotonin selective reuptake inhibitors; SNRIs, serotonin norepinephrine reuptake inhibitors; TCAs, tricyclic antidepressants; SD, standard deviation; YGTSS, Yale Global Tic Severity Rating Scale; CGI‐S, Clinical Global Impression of Severity; PUTS, Premonitory Urge for Tics Scale; RVBTRS, Rush Video‐Based Tic Rating Scale; ASRS, Adult ADHD Self‐Report Scale; Y‐BOCS, Yale‐Brown Obsessive‐Compulsive Scale; BAI, Beck Anxiety Inventory; BDI, Beck Depression Inventory.

### Computational Model Results

Figure S2 shows the simulated electric field magnitudes across all 6 regions for each of the included 7 participants. The interindividual ranges of simulated mean electric field magnitude were 0.1 to 0.17 V/m for the SMA mask, 0.1 to 0.19 V/m for SMA proper, and 0.1 to 0.19 V/m for pre‐SMA, suggesting that electric stimulation in the active arm targeted evenly both SMA proper and pre‐SMA of each hemisphere, in line with predictions when using 5 × 5‐cm conventional sponge pad tDCS.

### Primary Outcome

**FIG. 2:**
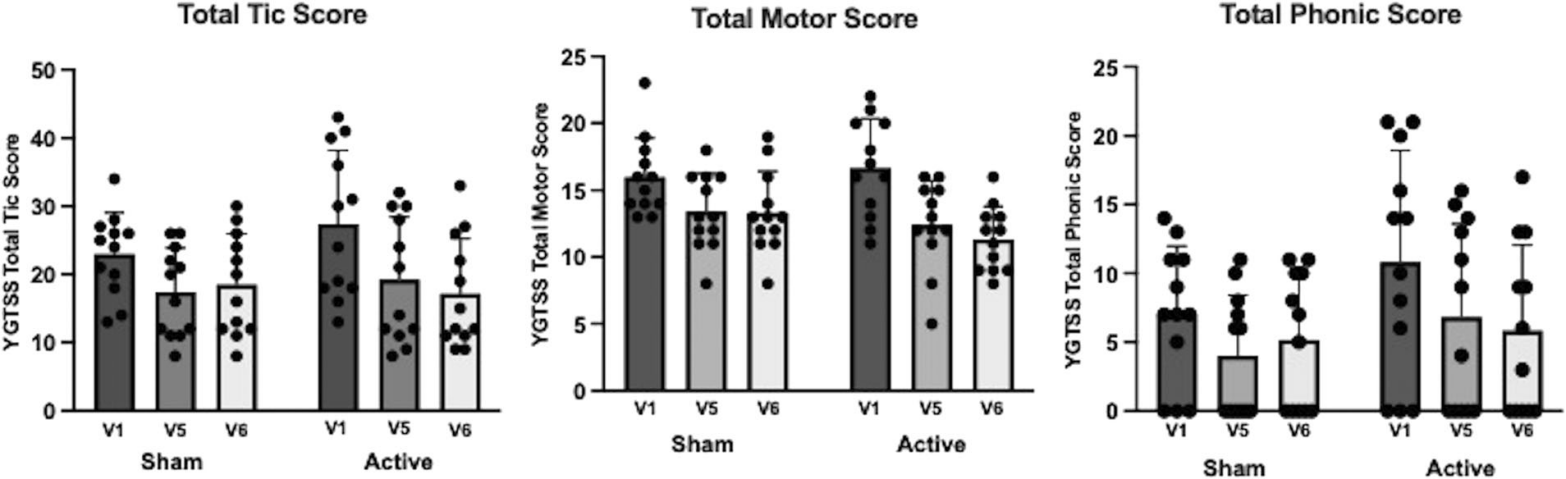
Yale Global Tic Severity Scale (YGTSS) Total Tic Severity (TTS) (left), total motor (center), and total phonic (right) subscores at visits 1 (V1), 5 (V5), and 6 (V6) in the sham and active stimulation groups (mean + standard deviation). Repeated‐measures analysis of variance (ANOVA) of YGTSS‐TTS score showed no main effect of treatment (F (1, 22) = 0.36, *P* = 0.56), a significant main effect of visit (F (1, 22) = 24.32, *P* < 0.0001), and only a trend toward a significant treatment × visit interaction (F (1, 22) = 4.90, *P* = 0.06). For the motor tic subscore, there was a trend toward a significant effect of treatment (F (1, 22) = 3.92, *P* = 0.06), a main effect of visit (F (1, 22) = 40.16, *P* < 0.0001), and a main effect of the treatment × visit interaction (F (1, 22) = 3.9, *P* = 0.03). Post hoc between‐group comparisons showed a significantly greater subscore difference from visits 1 to 6 in the active arm (*P* = 0.031) but not from visits 1 to 5 (*P* = 0.24). For the phonic tic subscore, the repeated‐measures ANOVA showed only a main effect of visit (F (1, 22) = 10.16, *P* = 0.0002) but no significant effect of treatment (F (1, 22) = 0.03, *P* = 0.86) or of the treatment × visit interaction (F (1, 22) = 1.67, *P* = 0.2).

The mean change in the YGTSS‐TTS score from visits 1 to 6 was 10.25 (SD = 7.90) in the active arm and 4.50 (SD = 6.57) in the sham arm. Repeated‐measures ANOVA of YGTSS‐TTS score showed no main effect of treatment (F (1, 22) = 0.36, *P* = 0.56), a significant main effect of visit (F (1, 22) = 24.32, *P* < 0.0001), and only a trend toward a significant treatment × visit interaction (F (1, 22) = 4.90, *P* = 0.06; Table 2; Fig. 2, left).

**TABLE 2:** Results of the repeated‐measures analysis of variance for primary and secondary outcome measures related to tics, detailing the main effects of treatment, visit, and the treatment × visit interaction; the between‐group comparisons of the change from visits 1 to 6 for premonitory urges and severity of comorbidities

Clinical severity outcome measure		F (DFn, DFd)	*P*‐value	Between groups comparison	
				Visits 1–5	Visits 1–6
YGTSS Total Tic Severity score	Treatment	F (1, 22) = 0.36	*P* = 0.56	Active Contrast: −8.25; ***P*** < **0.001** Sham Contrast: −5.58; ***P*** = **0.001**	Active Contrast: −10.33; ***P* < 0.001** Sham Contrast: −4.5; ***P*** = **0.007**
	Visit	F (1, 22) = 24.32	***P*** < **0.0001**		
	Treatment × visit	F (1, 22) = 3.02	*P* = 0.059		
YGTSS Total Motor Tic score	Treatment	F (1, 22) = 3.92	*P* = 0.06	Active Contrast: −4.25; ***P*** < **0.001** Sham Contrast: −2.58; ***P*** < **0.001**	Active Contrast: −5.33; ***P*** < **0.001** Sham Contrast: −2.67; ***P*** < **0.001**
	Visit	F (1, 22) = 40.16	***P*** < **0.0001**		
	Treatment × visit	F (1, 22) = 3.9	***P*** = **0.03**		
YGTSS Total Phonic Tic score	Treatment	F (1, 22) = 0.03	*P* = 0.86	Active Contrast: −4; ***P*** = **0.001** Sham Contrast: −3; ***P*** = **0.02**	Active Contrast: −5; ***P*** < **0.001** Sham Contrast: −1.83; *P* = 0.14
	Visit	F (1, 22) = 10.16	***P*** = **0.0002**		
	Treatment × visit	F (1, 22) = 1.67	*P* = 0.2		
CGI‐S	Treatment	F (1, 22) < 0.01	*P* = 1.0	Active Contrast: −0.75; ***P*** = **0.001** Sham Contrast: −0.5; ***P*** = **0.006**	Active Contrast: −1.08; ***P*** < **0.001** Sham Contrast: −0.5; ***P*** = **0.006**
	Visit	F (1, 22) = 21.36	***P*** < **0.0001**		
	Treatment × visit	F (1, 22) = 2.63	*P* = 0.08		
PUTS	N/A			N/A	Active Contrast: −4; ***P*** = **0.001** Sham Contrast: −0.1; *P* = 0.94
RVBTRS	Treatment	F (1, 19) = 0.07	*P* = 0.8	Active Contrast: −0.97; *P* = 0.19 Sham Contrast: −2.11; ***P*** = **0.01**	Active Contrast: −0.64; *P* = 0.39 Sham Contrast: −0.89; *P* = 0.36
	Visit	F (1, 14) = 3.87	***P*** = **0.03**		
	Treatment × visit	F (1, 14) = 0.57	*P* = 0.57		
Tic count	Treatment	F (1, 19) = 0.64	*P* = 0.43	Active Contrast: −9.6; ***P*** = **0.03** Sham Contrast: −13.89; ***P*** = **0.004**	Active Contrast: −6.74; *P* = 0.12 Sham Contrast: −5.11; *P* = 0.37
	Visit	F (1, 14) = 6.57	***P*** = **0.004**		
	Treatment × visit	F (1, 14) = 0.39	*P* = 0.68		
Inhibition potency	Treatment	F (1, 19) = 0.60	*P* = 0.45	Active Contrast: −0.14; *P* = 0.11 Sham Contrast: −0.09; *P* = 0.38	Active Contrast: −0.06; *P* = 0.49 Sham Contrast: 0.17; *P* = 0.13
	Visit	F (1, 13) = 4.81	***P*** = **0.015**		
	Treatment × visit	F (1, 13) = 0.32	*P* = 073		
ASRS	N/A			N/A	*P* = 0.36
Y‐BOCS	N/A			N/A	*P* = 0.50
BDI	N/A			N/A	*P* = 0.11
BAI	N/A			N/A	*P* = 0.11

Significant effects are indicated in bold font.

Abbreviations: YGTSS, Yale Global Tic Severity Rating Scale; CGI‐S, Clinical Global Impression of Severity; PUTS, Premonitory Urge for Tics Scale; RVBTRS, Rush Video‐Based Tic Rating Scale; ASRS, Adult ADHD Self‐Report Scale; Y‐BOCS, Yale‐Brown Obsessive‐Compulsive Scale; BDI, Beck Depression Inventory; BAI, Beck Anxiety Inventory; NA, not applicable for these outcome measures, which were collected only at baseline and visit 6, and analyzed for each arm using Bonferroni‐corrected 2‐tailed Student's *t*‐tests.

For the motor tic subscore, we observed a trend toward a significant effect of treatment (F (1, 22) = 3.92, *P* = 0.06), a main effect of visit (F (1, 22) = 40.16, *P* < 0.0001), and a main effect of the treatment × visit interaction (F (1, 22) = 3.9, *P* = 0.03). There was greater reduction in the motor tic subscore from visits 1 to 6 in the active arm (mean = 5.33, SD = 3.37) compared to the sham arm (mean = 2.67, SD = 2.19). Post hoc between‐group comparisons showed a significantly greater subscore difference from visits 1 to 6 in the active arm (*P* = 0.031) but not from visits 1 to 5 (*P* = 0.24) (Table 2; Fig. 2, center).

For the phonic tic subscore, the repeated‐measures ANOVA showed only a main effect of visit (F (1, 22) = 10.16, *P* = 0.0002) but no significant effect of treatment (F (1, 22) = 0.03, *P* = 0.86) or of the treatment × visit interaction (F (1, 22) = 1.67, *P =* 0.2; Table 2; Fig. 2, right).

### Secondary Outcomes

**FIG. 3:**
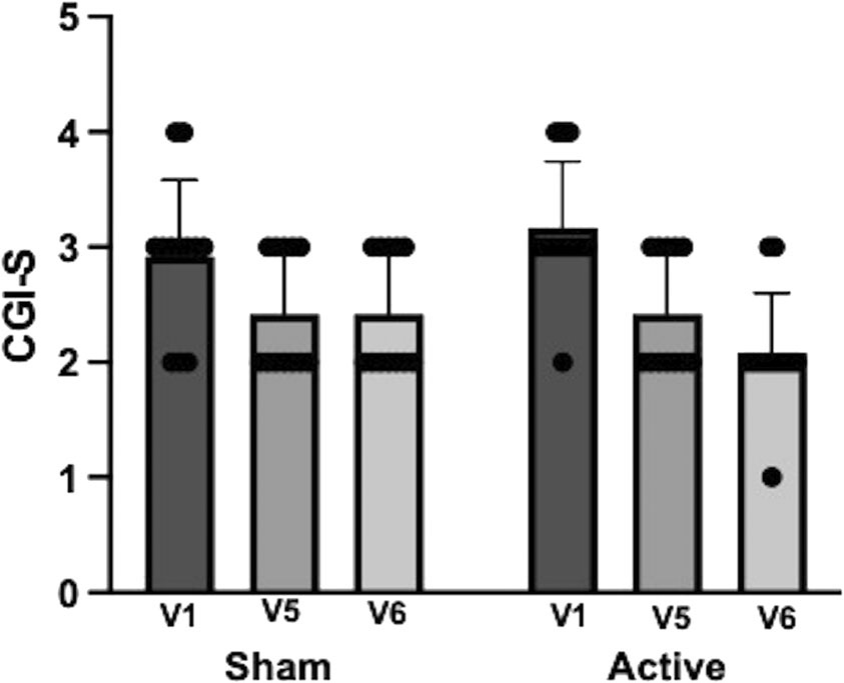
Clinical Global Impression of Severity (CGI‐S) (mean + standard deviation) at visits 1 (V1), 5 (V5), and 6 (V6) in the sham and active stimulation groups. The repeated‐measures ANOVA (analysis of variance) of CGI did not show any significant effect of treatment group or the interaction between treatment group and visit (F (1, 22) < 0.01, *P* = 1; F (1, 22) = 2.63, *P* = 0.08) but a significant main effect of visit (F (1, 22) = 21.36, *P* < 0.0001).

The repeated‐measures ANOVA of CGI did not show any significant effect of treatment group or the interaction between treatment group and visit (F (1, 22) < 0.01, *P* = 1; F (1, 22) = 2.63, *P* = 0.08) but a significant main effect of visit (F (1, 22) = 21.36, *P* < 0.0001; Table 2; Fig. 3).

**FIG. 4:**
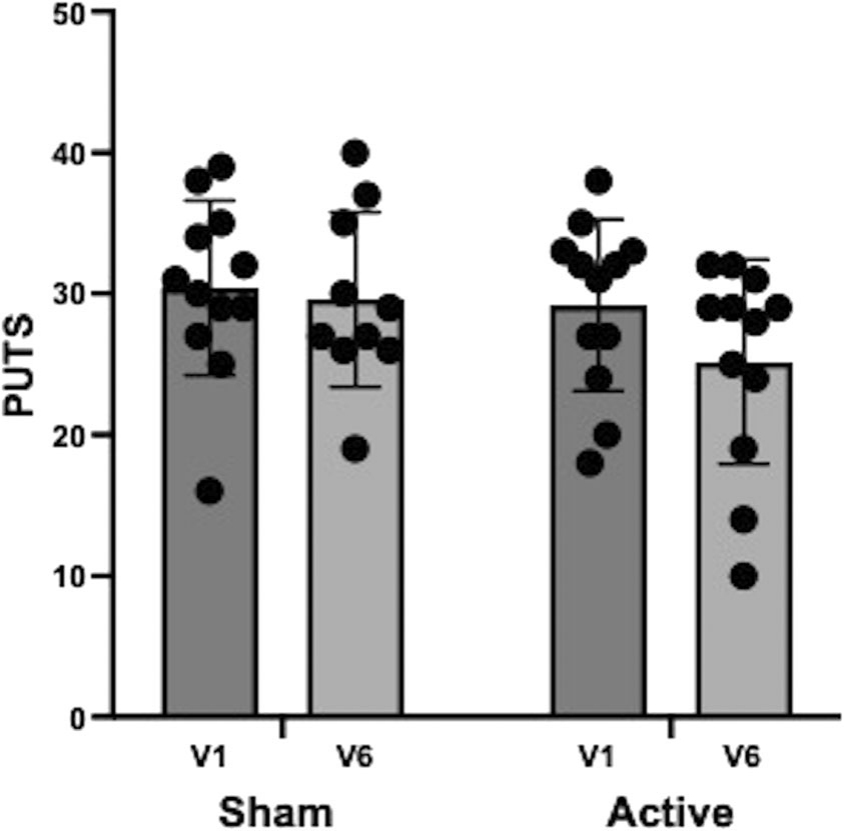
Premonitory Urge for Tics Scale (PUTS) (mean + standard deviation [SD]) at visits 1 (V1) and 6 (V6) in the sham and active stimulation arms. Student's *t*‐tests showed a significantly lower PUTS score at visit 6 compared to visit 1 in the active arm (mean = 4, SD = 4.92) with respect to the sham arm (mean = 0.1, SD = 3.035; *t* (20) = 2.18, *P* = 0.041).

Student's *t*‐tests showed a significantly lower PUTS score at visit 6 compared to visit 1 in the active arm (mean = 4, SD = 4.92) with respect to the sham arm (mean = 0.1, SD = 3.035; *t* (20) = 2.18, *P* = 0.041; Table 2; Fig. 4).

The mixed effect analysis of the RVBTRS score showed a main effect of visit (F (1, 14) = 3.87, *P* = 0.03) but not of treatment (F (1, 19) = 0.08, *P* = 0.8) or treatment × visit interaction (F (1, 14) = 0.57, *P* = 0.57; Table 2; Fig. S3). Similarly, the mixed effects analysis of tic count and IP showed only a significant main effect of visit but no significant effect of treatment or the treatment × visit interaction (Table 2; Fig. S3).

Two‐tailed Student's *t*‐tests showed no significant difference between active and sham arms in the score change from visits 1 to 6 in the ASRS (*P* = 0.36, *t* (19) = 0.93) and Y‐BOCS scores (*P* = 0.5, *t* (20) = 0.68). Similarly, no significant difference emerged with respect to the score differences between visits 1 and 6 for both BDI (*P* = 0.11, *t* (20) = 1.65) and BAI (*P* = 0.11, *t* (20) = 1.66) (Fig. S4).

### Tolerability

Nineteen participants (11 in the active arm) reported intra‐stimulation scalp paresthesia, usually within the first 2 min of stimulation, but no other intra‐stimulation side effects; the frequency of intra‐stimulation side effects did not significantly differ between the 2 arms (Fisher's exact test = 0.32). Side effects between stimulation sessions were uncommon: transient headache in 1 (active arm), excessive daytime sleepiness and fatigue in 2 (1 active and 1 sham), and transient early insomnia in 2 (1 active and 1 sham); the frequency of side effects between stimulation sessions did not significantly differ between the 2 arms (Fisher's exact test = 1).

## Discussion

To the best of our knowledge, this is the first double‐blind, sham‐controlled randomized trial of repeated sessions of tDCS for the treatment of tic severity in TS. We did not observe a significant effect of treatment on the YGTSS‐TTS score and only a trendwise effect of the interaction between time (visit) and type of stimulation. Analyzing motor and phonic tic subscores separately resulted in a significant effect of the “treatment × visit” interaction for motor, but not phonic, tic subscore, showing a beneficial effect of active stimulation 1 week after the end of the stimulation. Other secondary measures of tic severity (CGI‐S, RVBTRS, video‐based tic count) and ability to suppress tics did not yield a significant effect of stimulation. However, a significantly larger decrease in premonitory urge intensity 1 week after active stimulation compared to sham was observed, whereas attention deficit hyperactivity disorder (ADHD), depression, and anxiety did not yield an effect of stimulation.

Previous randomized controlled neuromodulation trials involving SMA in TS had mainly negative primary outcomes^15^, ^16^, ^19^ but some notable methodological differences from ours, including differences in participants and study duration. Landeros‐Weisenberger and colleagues^15^ compared active versus sham 1‐Hz rTMS over SMA in 20 adults with severe TS (15 treatment sessions over 3 weeks). There was no significant effect on YGTSS‐TTS score, but an open‐label 3‐week extension of active stimulation showed significant improvement from baseline in the 7 participants completing 6 weeks of active stimulation, suggesting a potential role of stimulation dosage. Another sham‐controlled trial^16^ of functional MRI (fMRI)‐navigated continuous θ burst magnetic stimulation over the SMA, applied in 8 sessions over 2 days in 12 participants (mean age: 13.5 years), did not show a significant between‐group difference in the YGTSS decrease 1 week after intervention. A single‐session, sham‐controlled trial of bilateral cathodal tDCS over SMA with 10 participants^19^ showed lower tic impairment scores lower after active stimulation. However, this single‐session study defined a video‐based rating instrument as primary outcome, did not provide specific subscores for motor and phonic tics, and assessed tic severity only once after stimulation.

Our study included patients with common comorbidities and medication use, representing naturalistically individuals with suboptimal control of tics with established tic‐suppressing treatments. Older adolescents and adults with TS, as included in our study, represent a subset of patients with more severe symptoms persisting beyond childhood,^39^ who might be better candidates for neurostimulation treatments.^5^ In addition to the YGTSS‐TTS score,^23^, ^40^ we separately analyzed motor and phonic subscores from the same instrument, given potential differences in neurobiological underpinnings between motor and vocal tics suggested by animal models that demonstrate a greater role of cortico‐basal ganglia‐cerebellar networks (encompassing SMA) in motor tics and a higher relevance of limbic networks (including the anterior cingulate cortex) for vocal tics.^41^, ^42^ Moreover, participants in our study were homogeneously engaged in active tic suppression during stimulation, thus potentially generating a more standardized brain state compared to a resting brain state, as tic suppression can influence motor cortex excitability.^43^, ^44^ Active stimulation and tic suppression may have resulted in a synergistic effect enhancing active stimulation. This is supported by the concept of activity selectivity, which suggests that already active brain networks may be more susceptible to modulation by tDCS.^45^

The observed reduction in the PUTS score after active stimulation may be related to the role of the SMA, together with the cingulate cortex and insula, in generating premonitory urges.^46^ The lack of effect on IP may instead reflect distinct neurobiological bases for tic urge and inhibition. Both phenomena engage the SMA but in the context of different networks. Neuroimaging studies indicate an association between urges and a network including insula, prefrontal cortex, anterior cingulate cortex, and SMA, whereas tic inhibition is driven by a functional network involving basal ganglia, inferior frontal gyrus, and pre‐SMA.^9^, ^47^ The relatively low scores on the CGI‐S at visit 1 and the relatively short interval between visits 1 and 6 may have limited our ability to detect intervention‐dependent changes. Unlike YGTSS, tic counts were not affected by active tDCS, suggesting that this intervention might affect other domains beyond frequency, for example, intensity, complexity, and interference, all included in the YGTSS severity score. Overall, the constructs of these 2 measures do differ: tic count measures tic number and frequency objectively and may be more influenced by the experimental setting of the video recording, whereas the YGTSS is more comprehensive, including subjective domains like tic intensity and interference, and evaluates tics over the preceding week.

The lack of effects on comorbid disorders requires specific consideration. Our study was not powered to detect a significant effect on these comorbidities, as their frequency was lower than 50% of the study sample. The pre‐SMA influences inhibitory control of ongoing actions and can be hyperactive in Obsessive‐Compulsive Disorder (OCD).^48^ However, recent evidence^49^ highlighted the importance of stimulation dosage on clinical efficacy of tDCS targeting the prefrontal‐SMA network, with higher efficacy using 2‐mA stimulation within an intensified protocol. Besides its low prevalence in our sample, the lack of effect on ADHD may be due to the selected target and polarity, given that prefrontal anodal tDCS has been reported as the most efficient protocol in ADHD. Finally, the instruments rating ADHD, depression, and anxiety may not be sensitive enough to detect meaningful changes after 1 week, and the stimulation area was not plausible to predict effects on depression and anxiety.

An important limitation of our study is its relatively low sample size. Further, although participants met the predefined minimum severity criteria for TS at screening, baseline severity at visit 1 was somewhat lower, potentially leading to a floor effect, especially for phonic tics. The lack of a physiological outcome measure is another limitation. A previous rTMS study in TS showed no effect on YGTSS but identified a significant reduction in fMRI‐derived blood oxygen level‐dependent signal during finger tapping in the SMA and bilateral M1 regions in the active stimulation group.^16^ This highlights potential physiological changes not captured by clinical scores, which prevents us from providing an evidence‐supported mechanistic explanation for the effect of stimulation on clinical outcomes. Furthermore, our study design was limited to cathodal tDCS. The relationship between direction of effect and stimulation polarity does not always follow the dichotomy of cathodal = inhibitory and anodal = excitatory.^18^ More work is needed to describe the effect of both polarities on physiological markers of SMA and of its functional connectivity.

In conclusion, this double‐blind, sham‐controlled RCT provides preliminary evidence that tDCS over the SMA in TS may reduce motor tic severity. Future studies with larger sample size, exploring higher stimulation intensity and session numbers, are needed to replicate and expand these findings. Whether multi‐electrode approaches of tDCS over the SMA yields greater efficacy than conventional tDCS is also worth exploring. Further studies could incorporate patient‐reported outcome measures, such as the Motor tic, Obsession and Compulsion, and Vocal tic Evaluation Scale (MOVES)^50^ and health‐related quality‐of‐life instruments such as the Gilles de la Tourette syndrome Quality‐of‐Life scale.^51^ Finally, coupling clinical scales with physiological markers of functional changes at a target or network level would provide a mechanistic basis for the observed effect on motor tic scores.

## Author Roles

(1) Research project: A. Conception, B. Organization, C. Execution; (2) Statistical analysis: A. Design, B. Execution, C. Review and critique; (3) Manuscript preparation: A. Writing of the first draft, B. Review and critique.

Y.M.: 1C, 2A, 2B, 3A

N.S.: 1C, 3B

L.S.G.: 1A, 1B, 2B, 3B

J.A.A.: 1C, 3B

M.A.N.: 1A, 2C, 3B

C.M.V.: 1A, 2C, 3B

T.M.P.: 1B, 3B

D.M.: 1A, 1B, 1C, 2A, 2C, 3B

## Disclosures

**Ethical Compliance Statement:** The University of Calgary's Research Ethics Board approved the study (REB17‐1615), and all work was carried out in accordance with the ethical standards specified in the 1964 Declaration of Helsinki. All participants provided written informed consent; parental consent and mature minor assent were sought for participants aged 16 or 17. We confirm that we have read the journal's position on issues involved in ethical publication and affirm that this work is consistent with those guidelines.

**Funding Sources and Conflicts of Interest:** This study was supported by institutional funding from the Department of Clinical Neurosciences, University of Calgary, awarded to Dr. Davide Martino. The authors declare that there are no conflicts of interest relevant to this work.

**Financial Disclosures for the Previous 12 Months:** Dr. Yasamin Mahjoub has no additional disclosures to report. Dr. Natalia Szejko is supported by an American Academy of Neurology Fellowship grant, the American Brain Foundation, and Tourette Association as well as a Polish Ministry of Health and travel grant from the International Parkinson's and Movement Disorders Society. Dr. Liu Shi Gan has no additional disclosures to report. Ms. Janet Adesewa Adeoti has no additional disclosures to report. Dr. Michael A. Nitsche is a member of the Scientific Advisory Boards of Neuroelectrics and Precisis. Dr. Carmelo M. Vicario has no additional disclosures to report. Dr. Tamara M. Pringsheim is supported by research grants from the Canadian Institutes of Health Research and the Azrieli Accelerator. Dr. Davide Martino has received consultancy fees from Roche, and honoraria as speaker from AbbVie, Merz Pharmaceuticals, the Dystonia Medical Research Foundation Canada, the International Parkinson's Movement Disorders Society, and the Canadian Movement Disorders Society. He receives honoraria as scientific advisor for the Ministry of University and Research of Italy. He receives royalties from Springer‐Verlag and Oxford University Press. He has received research grants from the Neuroscience, Rehabilitation and Vision Strategic Clinical Network of the Alberta Health Services, the National Institutes of Health (Dystonia Coalition), the Dystonia Medical Research Foundation (DMRF) USA, DMRF Canada, the National Spasmodic Torticollis Association (NSTA) Sacramento Chapter in Memory of Howard Thiel, the Canadian Institutes of Health Research, the Parkinson Foundation, the Azrieli Foundation, and the Calgary Parkinson's Research Initiative (CaPRI). He is a site principal investigator for a clinical trial for UCB and for an observational study supported by MRM Health NV and Nimble Science. Dr. Martino is chair of the Research Committee of the Board of the charity Parkinson's Association of Alberta.

## Supporting information


**Figure S1.** tDCS (transcranial direct current stimulation) current modeling was performed using the ROAST (Realistic vOlumetric‐Approach‐based Simulator for Transcranial electric stimulation) software package. (**A**) Mean electric field magnitude was calculated in 6 regions of interest in the simulated models: (i) left and right SMA (supplementary motor area) mask (in blue), with its location based on the AAL3 atlas; (ii) left and right SMA proper, with a 5‐mm‐radius sphere region of interest (ROI) centered at the MNI (Montreal Neurological Institute) coordinates of −3, −11, 61 and 3, –11, 61, respectively (in red); and (iii) left and right pre‐SMA, with a 5‐mm‐radius sphere ROI with the MNI coordinates of −4, 3, 59 and 4, 3, 59, respectively (in green). (**B**) Exemplary figures of the tDCS montage and simulation results in 1 subject. Virtual electrodes were placed on the model simulating the tDCS montage, with the cathode located 5 mm left or right to FCz and the anode over the contralateral mastoid. The gray circle shows the electric field magnitude at MNI coordinate −3, −11, 61.

**Figure S2.** The mean electric field magnitude calculated in 6 regions of interest in each of the 7 subjects receiving real stimulation, with the error bars representing the standard deviation.

**Figure S3.** Mean change (±SD [standard deviation]) in (**A**) the Rush Video‐Based Tic Rating Scale (RVBTRS) score, (**B**) tic count, and (**C**) calculated inhibition potency between visits 1 and 5, and between visits 1 and 6, in the sham and active stimulation groups.

**Figure S4.** Scores (mean + standard deviation) in (**A**) the Adult ADHD Self‐Report Scale (ASRS), (**B**) Yale‐Brown Obsessive‐Compulsive Scale (Y‐BOCS), (**C**) Beck Depression Inventory (BDI), and (**D**) Beck Anxiety Inventory (BAI) at visits 1 (V1) and 6 (V6) in the sham and active stimulation groups.

**Data S1.** Additional details on the outcome measures for tics and premonitory urges.

## Data Availability

The data that support the findings of this study are available on request from the corresponding author. The data are not publicly available due to privacy or ethical restrictions.
