# Supplementary figures and images for: Randomized Controlled Trial of Transcranial Direct Current Stimulation over the Supplementary Motor Area in Tourette Syndrome
Source: Mov Disord Clin Pract. 2024 Nov 29;12(3):313–24. doi: 10.1002/mdc3.14285 (PMC11952956; doi:10.1002/mdc3.14285)

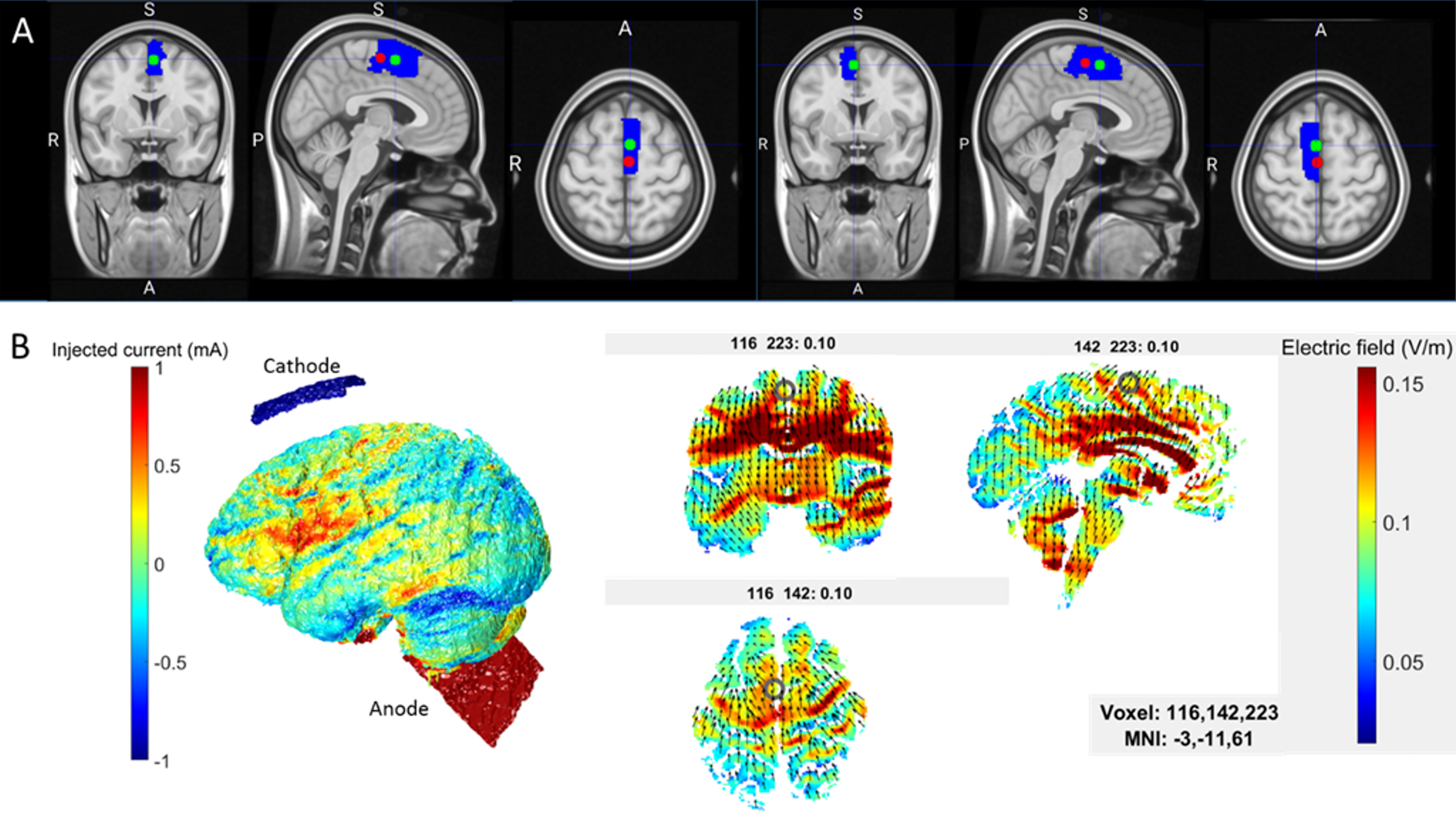

Supplement: Supplementary file 1 — Figure S1. tDCS (transcranial direct current stimulation) current modeling was performed using the ROAST (Realistic vOlumetric‐Approach‐based Simulator for Transcranial electric stimulation) software package. (A) Mean electric field magnitude was calculated in 6 regions of interest in the simulated models: (i) left and right SMA (supplementary motor area) mask (in blue), with its location based on the AAL3 atlas; (ii) left and right SMA proper, with a 5‐mm‐radius sphere region of interest (ROI) centered at the MNI (Montreal Neurological Institute) coordinates of −3, −11, 61 and 3, –11, 61, respectively (in red); and (iii) left and right pre‐SMA, with a 5‐mm‐radius sphere ROI with the MNI coordinates of −4, 3, 59 and 4, 3, 59, respectively (in green). (B) Exemplary figures of the tDCS montage and simulation results in 1 subject. Virtual electrodes were placed on the model simulating the tDCS montage, with the cathode located 5 mm left or right to FCz and the anode over the contralateral mastoid. The gray circle shows the electric field magnitude at MNI coordinate −3, −11, 61. [file MDC3-12-313-s005.png]

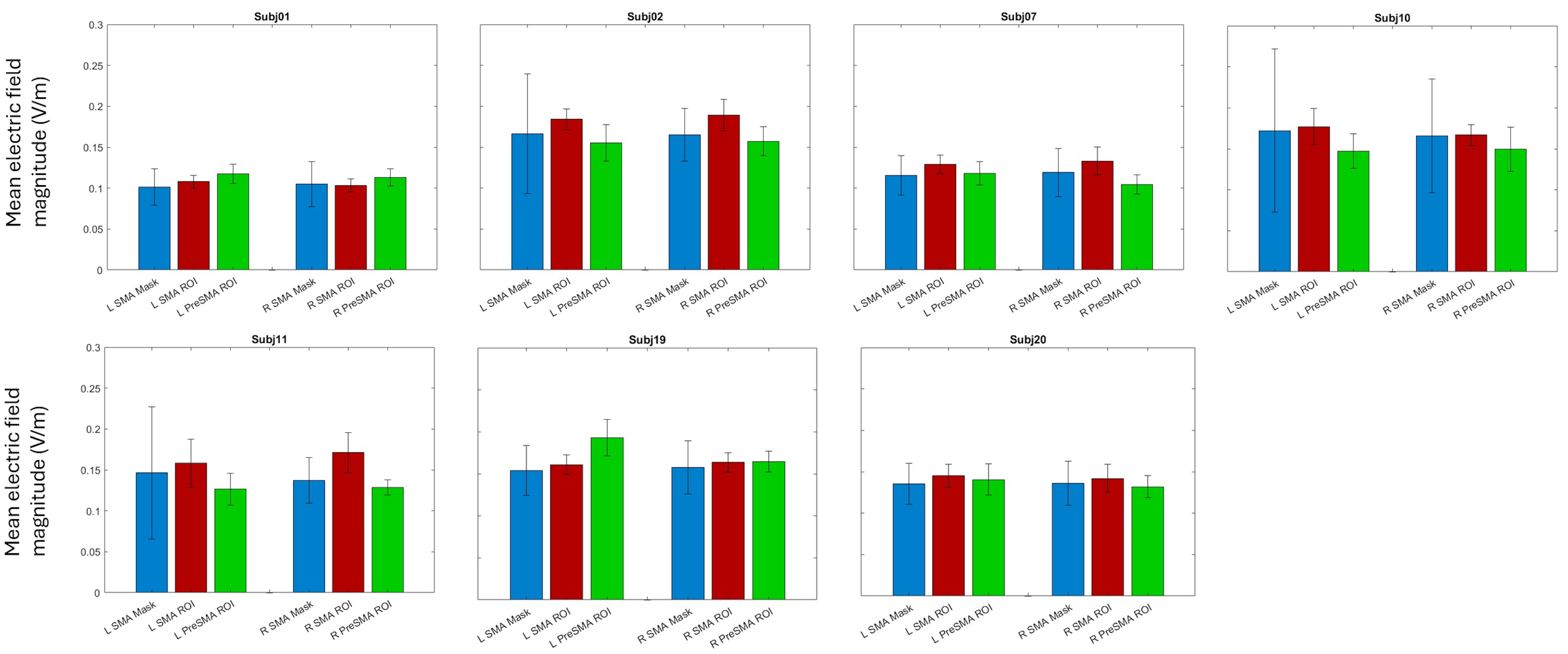

Supplement: Supplementary file 2 — Figure S2. The mean electric field magnitude calculated in 6 regions of interest in each of the 7 subjects receiving real stimulation, with the error bars representing the standard deviation. [file MDC3-12-313-s003.png]

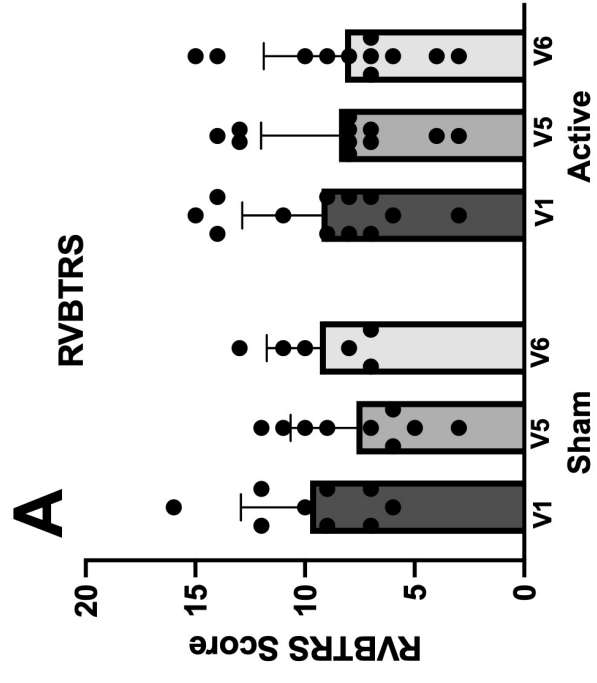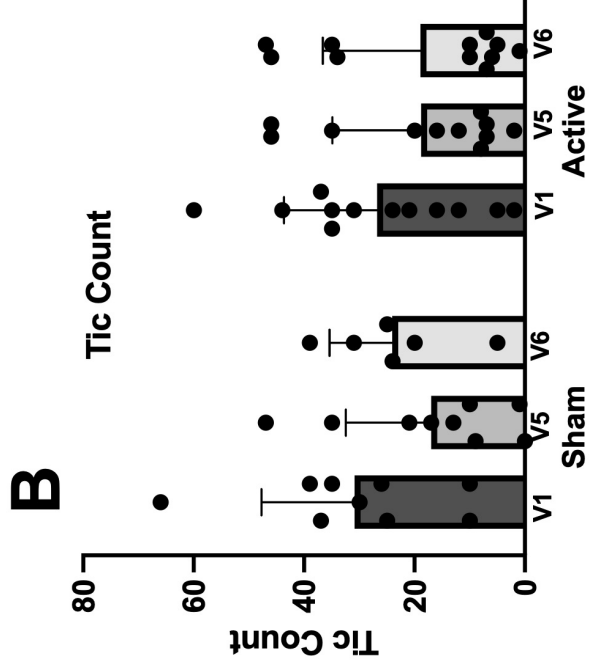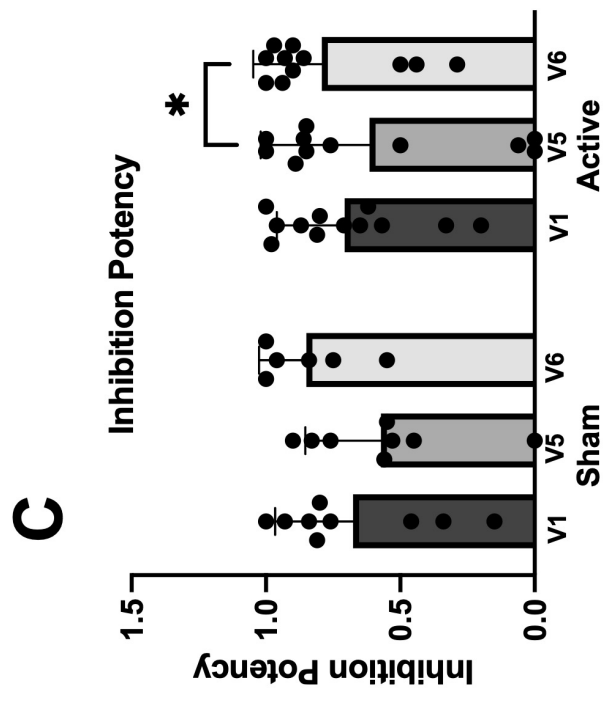

Supplement: Supplementary file 3 — Figure S3. Mean change (±SD [standard deviation]) in (A) the Rush Video‐Based Tic Rating Scale (RVBTRS) score, (B) tic count, and (C) calculated inhibition potency between visits 1 and 5, and between visits 1 and 6, in the sham and active stimulation groups. [file MDC3-12-313-s002.pdf]

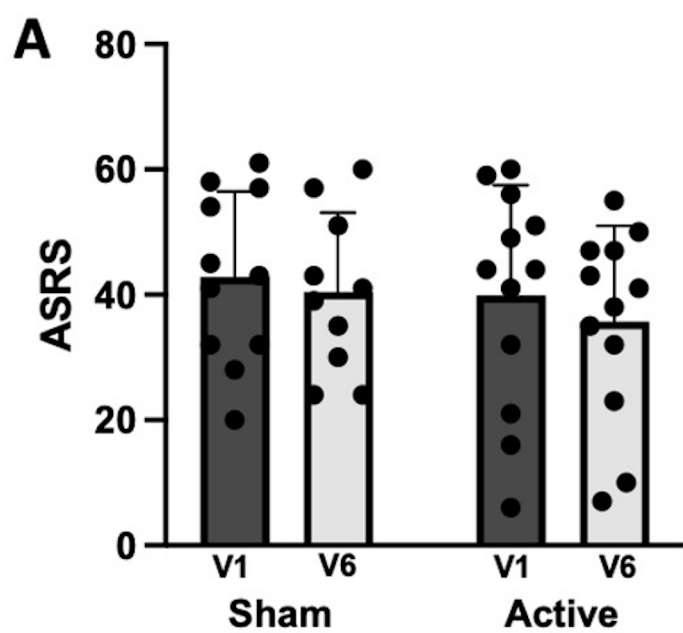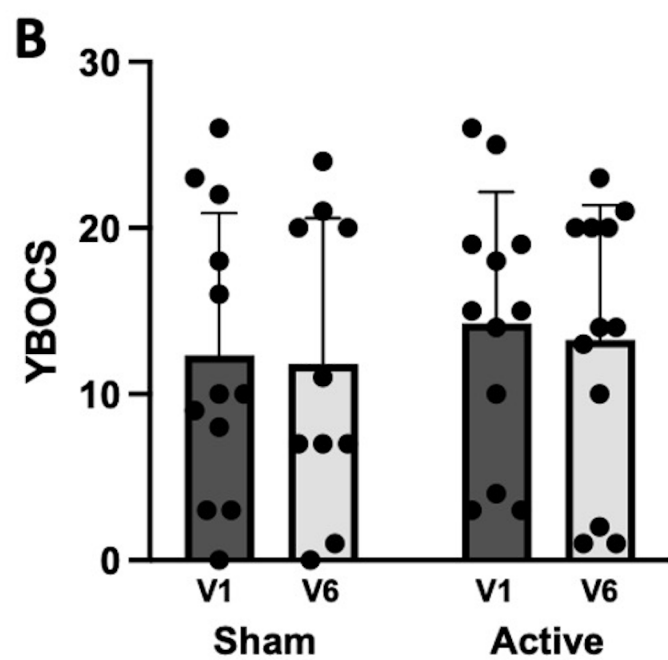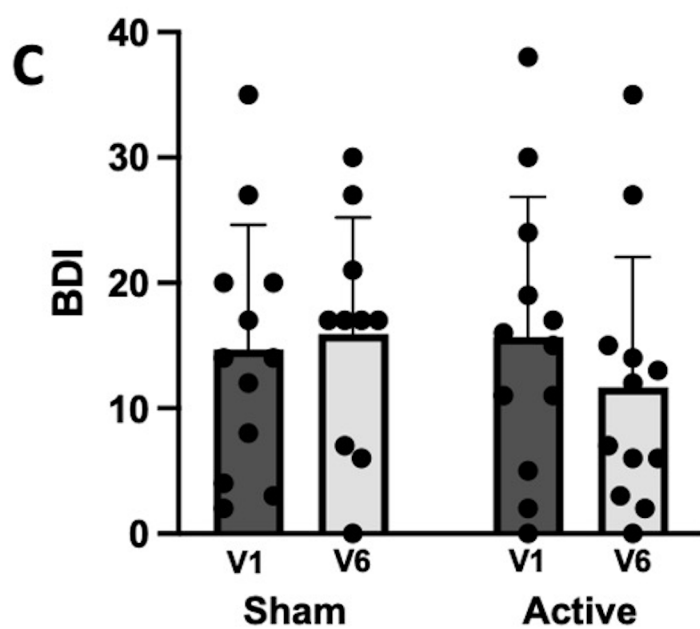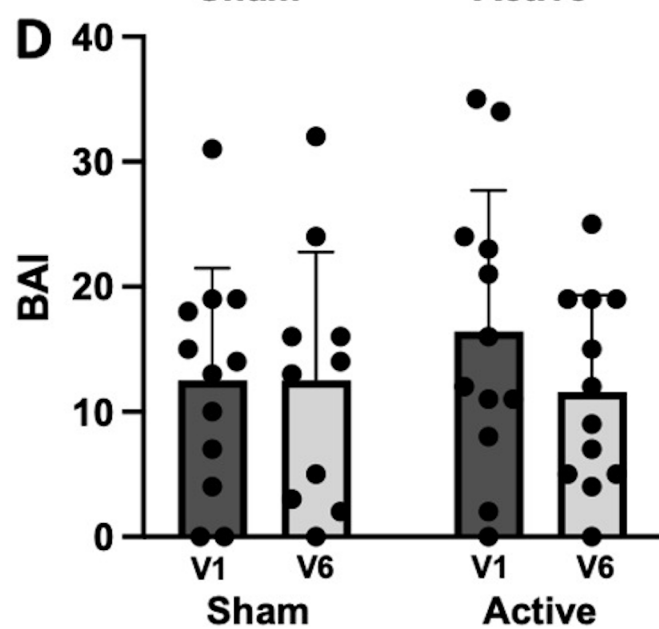

Supplement: Supplementary file 4 — Figure S4. Scores (mean + standard deviation) in (A) the Adult ADHD Self‐Report Scale (ASRS), (B) Yale‐Brown Obsessive‐Compulsive Scale (Y‐BOCS), (C) Beck Depression Inventory (BDI), and (D) Beck Anxiety Inventory (BAI) at visits 1 (V1) and 6 (V6) in the sham and active stimulation groups. [file MDC3-12-313-s004.pdf]
