# Supplementary material for: Randomized Controlled Trial of Transcranial Direct Current Stimulation over the Supplementary Motor Area in Tourette Syndrome
Source: Mov Disord Clin Pract. 2024 Nov 29;12(3):313–24. doi: 10.1002/mdc3.14285 (PMC11952956; doi:10.1002/mdc3.14285)
Supplement: Supplementary file 5 — Data S1. Additional details on the outcome measures for tics and premonitory urges. [file MDC3-12-313-s001.docx]

**Supplementary File**

The Yale Global Tic Severity Scale (YGTSS) is the most widely used recommended clinical assessment scale in TS research. It requires an experienced clinician using all available information to rate motor and phonic tic severity during the previous week. Five dimensions (number, frequency, intensity, complexity, and interference) of motor and phonic tics are rated separately on a 6-point Likert-type scale (0-5), with each point anchored to descriptive statements (score range 0-50). The YGTSS has very good internal consistency of the motor tic, phonic tic, and total tic severity sub-score, excellent inter-rater reliability, moderate-to-strong correlation with convergent scales, and strong divergent validity with respect to scales rating ADHD, OCD, aggressiveness, depression and anxiety.^28^.

The Rush Video-Based Tic Rating Scale (RVBTRS) rates tics using a video-based filming protocol that involves a 10-min film; patients are recorded with two body views and under two conditions (relaxed with the examiner in the room and relaxed with the patient alone). The scoring method includes five domains of tic disability (number of body areas, motor and phonic tic frequency, motor, and phonic tic severity), adopting a 5-point Likert rating (0-4) with fixed anchor points for each domain.

The Premonitory Urge for Tics Scale (PUTS) is a unidimensional scale including nine statements that are rated using four generic anchor points (“not at all true”, “a little true”, “pretty much true”, “very much true”), and results in a score ranging from 0-27.
